# Effects of L-serine ingestion on human sleep

**DOI:** 10.1186/2193-1801-3-456

**Published:** 2014-08-22

**Authors:** Yukihiko Ito, Satomi Takahashi, Manzhen Shen, Kohji Yamaguchi, Makoto Satoh

**Affiliations:** Central Research Laboratory, FANCL CORPORATION, Totsuka-ku, Yokohama, Kanagawa, 244-0806 Japan; Sleep Medicine, University of Tsukuba, Tennnodai, Tsukuba, Ibaraki, 305-8575 Japan

**Keywords:** Human sleep, L-serine, Amino acid

## Abstract

To investigate the effects of L-serine intake on human sleep, we conducted two randomized double-blinded crossover studies. In Study 1, healthy subjects who were dissatisfied with their sleep were given L-serine or a placebo 30 min before going to bed. After waking the next morning, subjective sleep quality was rated using the Ogri-Shirakawa-Azumi subjective sleep rating scale. In Study 2, subjective sleep quality was rated using the St. Mary’s Hospital sleep questionnaire, and objective parameters, including sleep initiation time, number of nighttime awakenings, and hours of sleep, were evaluated using actigraphy. In Study 1, factors related to “sleep initiation” and “sleep maintenance” during the L-serine intake period were significantly improved compared to the placebo intake period (p = 0.02 and p = 0.008, respectively). In Study 2, scores for “How well did you sleep last night?” and “How satisfied were you with last night’s sleep?” were significantly better during L-serine intake compared to placebo (p = 0.04 and p = 0.03, respectively). Subjective evaluation of sleep quality on waking was thus improved. In addition, objective evaluation using actigraphy showed that the “number of nighttime awakenings” tended to be decreased (p = 0.08). These findings suggest that intake of L-serine before going to bed may improve human sleep.

## Background

Sleep is a basic life process that greatly affects human health. The effects of sleep disturbance or deprivation on the brain, mind and body include not only hypobulia and depression, but also effects potentially leading to hypertension and obesity (Gangwisch et al. 2005), thus impairing human quality of life. According to a World Health Organization study, 1 of every 2 persons with insomnia develops some illness other than sleep disturbance within 1 year and requires medical care (Ustün et al. 1995). The number of individuals suffering from sleep difficulties because of changes in living environment continues to increase. In a survey on the incidence of insomnia, about 20% reported experiencing some difficulty sleeping, and this figure was 30% in elderly persons (Ministry of Health, Labour and Welfare Japan 2008; Zhdanova et al. 2001). Management of insomnia has thus become a social issue.

L-serine is a precursor of other amino acids such as glycine and L-cysteine, and of cell membrane lipids such as phospholipids and sphingolipids. L-serine plays an extensive role in protein synthesis and intracellular metabolism. In knockout mice in whom 3-phosphoglycerate dehydrogenase (PHGDH) in the L-serine synthetic pathway is inactivated, abnormal brain morphogenesis, including microcephaly and absence of specific regions, and brain dysfunction occurs (Yoshida et al. 1980). PHGDH deficiency in humans causes neuropathy and postnatal microcephaly (Pepplinkhuizen et al. 1980; de Koning et al. 2004). This postnatal microcephaly can be improved by L-serine administration during pregnancy (de Koning et al. 2004). Such reports show that L-serine plays an important role in central nervous system (CNS) morphogenesis and function.

Social isolation stress in neonatal chicks on removal from their flock is associated with increased active wakefulness and vocalization (Panksepp et al. 1981; Sahley et al. 1980; Feltenstein et al. 2003). L-serine administration in this model reduces locomotor activity and vocalization, and increases sleeping posture time (sitting motionless with head drooped) (Koutoku et al. 2005; Asechi et al. 2006; Asechi et al. 2008; Shigemi et al. 2010). However, the effects of L-serine on human sleep have not been reported. We therefore conducted two studies to clarify the effects on human sleep of L-serine intake before going to bed.

## Subject and methods

Studies 1 and 2 were both randomized double-blinded crossover studies that included subjects who were dissatisfied with their sleep, mainly sleep latency and nocturnal awakening. Protocols for both studies were reviewed and approved by the institutional review board at FANCL Corp. before being conducted. All subjects were fully informed about the nature and methods of the studies, and informed consent was obtained in compliance with the Declaration of Helsinki.

Study 1 included 53 subjects. During the study period, subjects were not permitted to drink alcohol, stay out overnight, or use any medications or supplements that would affect sleep. They were instructed to maintain their usual eating and lifestyle habits. Each night for 4 consecutive days, 30 min before going to bed, the subjects ingested 3 g of L-serine powder (content: ≥98.5%) or a placebo powder (trehalose). The wash-out period was ≥3 days.

Subjective sleep quality was evaluated in each subject within 30 min after waking the next morning using the Ogri-Shirakawa-Azumi subjective sleep rating scale (OSA), which is used for qualitative evaluation of sleep (Oguri et al. 1985). Scores were calculated for 5 factors as described in a previous report from the results of the subject responses to each item, rated in 6 grades. Mean values for each 4-day period were used for analysis. Factor 1, “morning sleepiness”, related to feeling sleepiness on waking in the morning. Factor 2, “sleep maintenance”, related to whether the subject experienced waking during sleep. Factor 3, “morning vague anxiety”, related to vague anxiety on waking in the morning. Factor 4, “Satisfaction of sleep”, related to an intuitive feeling of sound sleep after waking. Finally, factor 5, “sleep initiation”, related to sleep onset. For all factors, better sleep quality was indicated by a higher score and poorer sleep quality by a lower score.

Study 2 included 9 subjects who consented to participate. The instructions given during the study period were the same as in Study 1. At night on 2 consecutive days, 30 min before going to bed, the subjects ingested 3 g of L-serine powder or a placebo powder. Subjective sleep quality was evaluated the next morning after waking using the St. Mary’s Hospital sleep questionnaire (SMH) (Ellis et al. 1981). The wash-out period was ≥3 days. In this study, the Japanese version of the SMH was used (Uchiyama et al. 1998). Mean values for the 2-day period were used for analysis. An actigraph (familymaicro; Ambulatory Monitoring, Inc., NY, USA) was attached to the non-dominant arm to record the number of body movements, with the results analyzed using AW-2 sleep analysis software (Ambulatory Monitoring, Inc., NY, USA), and sleep/wakefulness was distinguished using Cole's algorithm (Sadeh et al. 1995).

### Statistics

OSA and SMH scores were analyzed using Wilcoxon's signed rank test. Actigraphy data were analyzed using the paired t-test.

The level of significance was set at P < 0.05. The statistical analyses were performed using the statistical software program StatView for windows version 5.0 (SAS Institute., North Carolina).

## Results

**Figure 1:**
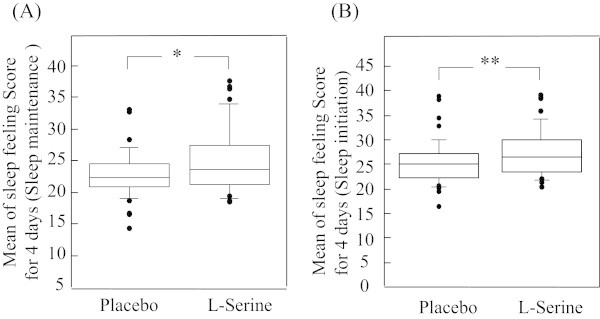
**Score of sleep feeling with OSA subjective sleep rating scale. (A)** Score of sleep feeling (Sleep maintenance). *Significant difference compared to Placebo by Wilcoxon's signed rank test; p < 0.02 (n = 45). **(B)** Score of sleep feeling (Sleep initiation). **Significant difference compared to Placebo by Wilcoxon's signed rank test; p < 0.008 (n = 45).

Among the 53 subjects in Study 1 who consented to participate, 8 withdrew during the study period. Reasons for withdrawal included use of anti-allergy medication for hay fever in 1 subject, a protocol deviation (drinking alcohol) in 1 subject, and requests to withdraw (change in work environment) in 6 subjects. Data were therefore analyzed from 45 subjects (10 men, 35 women), with a mean age of 35 ± 8 years (range, 26–59 years). Each of these subjects was dissatisfied in some way with their sleep quality. Based on the multiple responses, 23 had difficulty falling asleep, 33 experienced nighttime awakening, and 29 had early morning awakening. The “sleep maintenance” (L-serine, 24.9 vs. placebo, 22.8; p = 0.02) and “sleep initiation” (L-serine, 26.9 vs. placebo, 24.7; p = 0.008) factor scores were significantly better during L-serine intake compared with placebo (Figure 1). The “morning sleepiness” (L-serine, 24.1 vs. placebo, 22.8; p = 0.24), “morning vague anxiety” (L-serine, 26.7 vs. placebo, 24.9; p = 0.36), and “Satisfaction of sleep” (L-serine, 23.0 vs. placebo, 22.8; p = 0.75) factor scores showed no significant differences.

Among the 9 subjects in Study 2 who consented to participate, 2 withdrew during the study period. Reasons for withdrawal were a protocol deviation (staying out overnight) in 1 subject and “not feeling well” (lip numbness) during the placebo intake period in 1 subject. Data were therefore analyzed from 7 subjects (4 men, 3 women), with a mean age of 35 ± 8 years (range, 25–38 years). Eight of the original 9 subjects complained about difficulty falling asleep and nighttime awakening, and one complained only about nighttime awakening. One subject forgot to attach the actigraph, so actigraphy data were analyzed for 6 patients. Table 1 shows subjective sleep quality results based on the SMH questionnaire. Scores for question “How well did you sleep last night?” (p = 0.04) and question “How satisfied were you with last night’s sleep?” (p = 0.03) were significantly better during L-serine intake compared with placebo. Scores for question “How clear-headed did you feel after getting up this morning?” (p = 0.06) were tend to better during L-serine intake compared with placebo.

**Table 1:** **Score of St. Mary's hospital sleep questionnaire (Study 2)**

Subjective symptom after awake		Placebo	L-serine	P-value
Was your Sleep?	light < deep	3.4 ± 0.8	4.6 ± 1.7	0.09
How many times did you wake up?	time(s)	2.5 ± 2.0	1.9 ± 0.7	0.80
How well did you sleep last night?	badly < well	3.1 ± 0.8	4.2 ± 1.0	0.04
How clear-headed did you feel after getting up this morning?	still very drowsy < alert	2.1 ± 1.0	3.1 ± 1.1	0.06
How satisfied were you with last night's sleep?	unstisfied < satisfied	2.3 ± 0.7	3.3 ± 0.6	0.03
Were you troubled by waking early and being unable to get off to sleep	no < yes	1.2 ± 0.4	1.1 ± 0.2	0.18
How much difficulty did you have in getting off to sleep last night?	none or very little < difficult	1.5 ± 0.3	1.2 ± 0.3	0.15
How long did it take your to fall asleep last night.	mins	18.4 ± 14.9	12.9 ± 9.9	0.73

Values are means ± SD. n =7.

Significant difference compared to Placebo by Wilcoxon's signed rank test.

Subjective evaluation of sleep quality on waking in the morning was improved. No significant differences were seen for any other item. Table 2 shows the results for objective evaluation using actigraphy. The number of nighttime awakenings during L-serine intake tended to be decreased compared with placebo.

**Table 2:** **Score of actigraphy items (Study 2)**

Actigraphy item		Placebo	L-serine	P-value
Sleep latency	min	12.3 ± 6.0	7.2 ± 1.8	0.46
Sleeping time	min	360 ± 53	366 ± 38	0.56
Arousal time	min	21.6 ± 15.8	18.3 ± 17.6	0.72
Awake frequency	time(s)	8.1 ± 2.2	5.8 ± 3.1	0.08
Long wake episode	time(s)	2.9 ± 1.2	1.4 ± 3.1	0.38

Values are means ± SD. n =6.

Significant difference compared to Placebo by paired t-test.

## Discussion

The results of this study suggest that intake of L-serine before going to bed improves subjective sleep quality among individuals who are dissatisfied with their sleep. The results of objective evaluation using actigraphy also support these findings.

In Study 1, parameters related to nighttime awakening and sleep initiation on the OSA were significantly improved, but Study 2 found no significant improvements in the corresponding items. This may have been influenced by the smaller number of subjects in Study 2 and differences in the questionnaire used. Objective evaluation using actigraphy showed that nighttime awakenings tended to be decreased. In terms of distinguishing sleep/wakefulness, the concordance between actigraphy and polysomnography (PSG) in healthy individuals is ≥90%, and a high concordance of 78-85% has also been reported in patients with sleep disorders (Kushida et al. 2001). However, results from the questionnaires used in our study and actigraphy did not show changes in sleep depth or rapid eye movement (REM) sleep/non-REM sleep. Studies using PSG are needed to identify changes in sleep structure.

With regard to L-serine and reversible conversion to glycine in vivo, oral administration is reported to improve sleep in humans (Inagawa et al. 2006; Yamadera et al. 2007). Glycine is an inhibitory neurotransmitter like gamma-aminobutyric acid (GABA). Shigemi et al. investigated the effects of simultaneous administration of the GABAA receptor antagonist picrotoxin, the glycine receptor antagonist strychnine, and L-serine (Shigemi et al. 2008). They found that the hypnotic effects of L-serine were inhibited by the GABAA receptor antagonist picrotoxin, but not by strychnine. On the other hand, the hypnotic effects of glycine were inhibited by the glycine receptor antagonist strychnine. That report showed that the mechanism of action differs from that for glycine. However, whether a similar mechanism of action exists in humans is unknown from our study.

In a social isolation stress model in neonatal chicks, Furuse et al. compared L-serine-related phosphoserine, acetyl serine, lysophosphatidylserine, L-alanine, lysine, methionine, and tryptophan; and reported that L-serine had both hypnotic and anxiolytic effects (Koutoku et al. 2005; Asechi et al. 2008). In addition, D-serine, an optical isomer of L-serine, is present in the vertebrate brain, and particularly in mammals, is an N-methyl-D-aspartate (NMDA) receptor agonist. However, in the neonatal chick model of isolation stress, decreased locomotor activity and vocalization, as well as prolonged sleep-like behavior, were confirmed only with L-serine, while administration of D-serine had no effect (Asechi et al. 2006). Furthermore, measurement of plasma and cerebral cortical levels of L- and D-serine after oral administration in rats showed that L-serine levels increased in both plasma and the cerebral cortex, whereas D-serine did not (Tomonaga et al. 2012). The improvement in human sleep with L-serine observed in our study was thus probably not mediated by D-serine synthesis.

Subjects in our study took L-serine 30 min before going to bed. With oral administration of L-serine to rats, plasma L-serine levels peaked 30 min after administration, then decreased, reaching baseline levels within 10 h (Tomonaga et al. 2012). We have confirmed increased plasma L-serine levels in humans 30 min after L-serine ingestion (data not shown). These results suggest that the timing of L-serine administration in our study was appropriate. In addition, subjects in both Studies 1 and 2 felt refreshed and had no problems the next morning after taking L-serine. No hangover effects of drowsiness were reported the next day.

When taking sleep-improving drugs, resistance after long-term administration or rebound insomnia after discontinuation are frequently problematic. We have shown that when subjects who are dissatisfied with their sleep drink a beverage containing 3 g of L-serine on consecutive days for 1 month, the improvement in sleep quality persists even 1 month after starting administration (data not shown). Moreover, follow-up for 1 month after discontinuing L-serine showed no worsening of sleep quality compared to before administration. Instead, although the effects were diminished compared to during L-serine intake, improved sleep status tended to be maintained. These results indicate no resistance to the effects of L-serine and no problems with rebound insomnia.

In a study of neonatal chicks, L-serine prolonged the time of sleep-like behavior that had been shortened by social isolation stress (Koutoku et al. 2005; Asechi et al. 2006; Asechi et al. 2008; Shigemi et al. 2010). In our Study 1, stratified analysis of 27 subjects who experienced stress in their daily lives also showed a significant improvement in “sleep initiation” (p = 0.017). These results demonstrate that L-serine can also improve sleep among individuals suffering from stress.

Our findings suggest that L-serine improves sleep initiation and nighttime awakenings, resulting in improved feelings of having slept well when waking in the morning. L-serine may represent a good option for individuals who suffer from difficulty sleeping.

## Conclusion

The results of this study suggest that consecutive intake of L-serine is effective for individuals experiencing sleep difficulty. L-serine may be a good choice for most individuals affected by poor sleep.

## Abbreviations

PHGDH: 3-phosphoglycerate dehydrogenase
CNS: Central nervous system
OSA: Ogri-Shirakawa-Azumi subjective sleep rating scale
SMH: St. Mary’s Hospital sleep questionnaire
PSG: Polysomnography
REM: Rapid eye movement
GABA: Gamma-aminobutyric acid
NMDA: N-methyl-D-aspartate.
